# Hospital Meetings

**Published:** 1904-05-14

**Authors:** 


### HOSPITAL MEETINGS.

### THE HOME HOSPITALS ASSOCIATION.

The annual meeting of this Association was held on May 4th at Fitzroy House. A telegram of regret for unavoidable absence having been read from the President, the Duke of Northumberland, Sir Henry Burdett, the Vice-President, took the chair.

The Chairman, in moving the adoption of the report, said that the present was a very interesting meeting, because it was the first since the Home Hospital had been rebuilt. Last year those who had been associated with the management of this hospital for the past 25 years felt that by having conducted a hospital on rates just inclusive of actual cost and by making it pay, they had fulfilled the main purpose of the Association, which was to help forward the movement in favour of enforcing the right of every citizen of London to have medical treatment when he needed it, and to pay such a sum as he could afford. At the present time the great hospitals of London received patients, no matter to what class they belonged, without any payment whatever. The result of this was, as had been foreseen by this Association nearly thirty years ago, that while medical science had developed and improved, the hospital population of London had increased out of all proportion to the growth of the population. He ventured to say that this state of things ought not to go on, and he felt that the Home Hospital had proved that the hospital authorities could properly provide accommodation for paying patients at varying rates, and thus relieve, to a material extent, the drain on their resources, and at the same time give better care and attention to those who could pay towards the cost of their treatment in the hospital. After having completed a period of 25 years' work they would certainly not have taken upon themselves last year the responsibility of expending £12,000 on rebuilding had it not been strongly represented to them, by a large number of the leading consultants of London, that the existence of this hospital, reconstructed and made up to date into a perfect model hospital, was essential to the well-being of a large number of the better classes, because nowhere else in London could they receive the care they needed at a definite and fixed rate without fear of extortion. They had yielded to this wish and rebuilt the hospital, and had asked those who had benefited by it in the past, or who might do so in the future, to subscribe the necessary capital. The response to that appeal, however, had been far from satisfactory. They found themselves in the position of having to make up the sum of £8,000 towards the total cost of rebuilding. Letters had been received from some who had used the hospital refusing to subscribe on the ground that as the institution was provided by means of contributions, those who used it might be regarded as objects of charity. Sir Henry then read an extract from a speech made by the Bishop of Winchester, in which it was proved that the hospital was no more eleemosynary in principle than were all the great public schools, and went on to say that the committee had determined not to make any more appeals but to treat the matter on a business footing, and to raise the money in a business way, and repay it by means of a sinking fund accruing from the profits earned by the hospital. In the past year they had made a profit of some £1,500, so that they might hope to liquidate their debt on account of the new building in about five or six years.

During the past year 415 patients had been under treatment in the hospital, the average number of patients throughout the year being 26 and the average length of stay 21·84 days. Another satisfactory fact was that those 415 patients were under the medical care of 76 of the leading consultants of London. He was glad to welcome Mr. L. H. Smith as a member of the committee of management. He regretted very much to say that his son, Mr. Arthur Burdett, though better, was not likely to be able to take up again the post of honorary secretary, which had been filled by Mr. W. Page. The thanks of the committee were due to Miss Pearson, the lady superintendent, for her excellent services in placing the new hospital on a satisfactory basis, both administrative and financial. He also wished to pay a warm tribute to the treasurer, Mr. Frederick Cox, for his self-sacrifice in continuing his labours for the new hospital. There was no single man connected with the hospital to whom greater thanks were due than to Mr. Cox.

The motion was seconded by the treasurer, and the ordinary business having been transacted the meeting became a special one for the purpose of appointing Mr. Guy Greenwood Hammersley one of the trustees of the Association in place of the late Duke of Northumberland.

A vote of thanks to the chairman terminated the proceedings.

### HOSPITAL FOR EPILEPSY AND PARALYSIS.

The annual meeting of the Governors of this hospital was held on May 5th, Mr. Charles Drummond, the treasurer, being in the chair.

The report for 1903 was then read by the secretary, the chief feature of which was the visit of H.R.H. Princess Louise (Duchess of Argyll) to open the new buildings last June. The cost of the new hospital was over £25,000, and when completed it would provide accommodation for 60 beds. The admission of in-patients had been unavoidably postponed owing to the failure of the material for the flooring of the passages, etc., and the difficulty they had found in obtaining a suitable substitute. The expenditure for 1903 was, in consequence, no criterion as to what it would be in the future, but it was certain that if they wished to keep out of debt they would require an additional income of £3,000 per annum in subscriptions and donations. The committee appealed most earnestly for increased assistance towards obtaining this yearly sum. As the result of a further protest to the Borough Council the rating of the hospital had been reduced, but it was even now over £200, which was still quite disproportionate to the rating of all the other large hospitals in London. During the past year Miss Dorothy Ogle, their matron, had relinquished her post, for family reasons, and Miss Elizabeth Kirby had been appointed to fill her place. The extraordinary receipts for 1903 were only £394, as against £954 in 1902. They had received £350 from the King's Hospital Fund. The increase in attendances had been well maintained, the total for last year being over 11,000.

The Chairman then moved the adoption of the report, which was seconded by Captain Ellis.

